# Commentary: Influence of smartphone addiction proneness of young children on problematic behaviors and emotional intelligence: Mediating self-assessment effects of parents using smartphones

**DOI:** 10.3389/fpsyg.2019.00115

**Published:** 2019-02-18

**Authors:** Qin Ying Joanne Tan, Andree Hartanto, Wei Xing Toh, Hwajin Yang

**Affiliations:** Psychology, Singapore Management University, Singapore, Singapore

**Keywords:** smartphone overuse, preschoolers, parental assessment of smartphone use, children's problematic behaviors, emotional intelligence

The majority of studies on smartphone addiction have focused on adults and school-aged children or youth (e.g., Hartanto and Yang, 2016; Chung et al., 2018; Lee et al., 2018); few have investigated the impact of smartphone overuse during infancy and early childhood. Recently, Cho and Lee (2017) surveyed parents of children aged one to six and attempted to address this research gap in their article entitled “Influence of smartphone addiction proneness of young children on problematic behaviors and emotional intelligence: Mediating self-assessment effects of parents using smartphones.” Although the results are interesting, we would caution that they are preliminary because of the study's lack of theoretical grounding and empirical evidence for the proposed mediation model and notable methodological problems. Our primary goal is therefore to draw attention to an alternative conceptual model that elucidates the causal relationship between parents' smartphone use, children's smartphone addiction proneness, and their problematic behaviors. In addition, we discuss methodological issues with sampling methods and psychometric properties of measures and suggest further studies to address these concerns.

The main limitation of the study is that Cho and Lee's (2017) mediation model is logically flawed and lacks both theoretical and empirical support. Given that a simple mediation model depicts a causal relationship between independent and dependent variables via the inclusion of a mediator (Mathieu and Taylor, 2006), the authors argue (Figure 1A) that young children's smartphone addiction proneness increases parents' self-assessment of smartphone use (i.e., a mediator), which in turn affects children's problematic behaviors. Theoretically, however, the reverse direction of the relationship (from parents' smartphone use to young children's smartphone addiction proneness) is more plausible, notwithstanding the slim possibility that children's smartphone addiction proneness may in part cause changes in parents' smartphone use. This is because infants and young children have quite limited access to smartphones—primarily due to parental restrictions or low smartphone ownership among infants and young children—while parents' access to their own smartphones is relatively less restricted. More importantly, given that children largely model their behaviors on their parents (Bandura, 1977), parents' smartphone use habits likely have a more obvious impact on young children's smartphone addiction proneness. In line with this notion, a recent empirical study (Lauricella et al., 2015) found that parents' own screen time across four media platforms—television, computers, smartphones, and tablets—is the strongest predictor of screen time for children aged 0–8 years. More robust evidence for the causal impact has been reported in longitudinal studies that find a direct influence of parental media use on children's screen time (e.g., Carson et al., 2015; Abbott et al., 2016). In view of these findings, an alternative mediation model in which parents' self-assessed smartphone use affects children's smartphone addiction proneness, which in turn influences children's problematic behaviors (Figure 1B), appears to have strong logical appeal. Although Cho and Lee claim a causal relationship between children's smartphone proneness and behavioral problems via parents' smartphone use, their study's lack of theoretical grounding and empirical evidence for the link between the proposed variables and the cross-sectional and correlational nature of the study could invalidate their presumed mediation model (Mathieu and Taylor, 2006; Fiedler et al., 2011) and preclude any firm conclusions.

**Figure 1:**
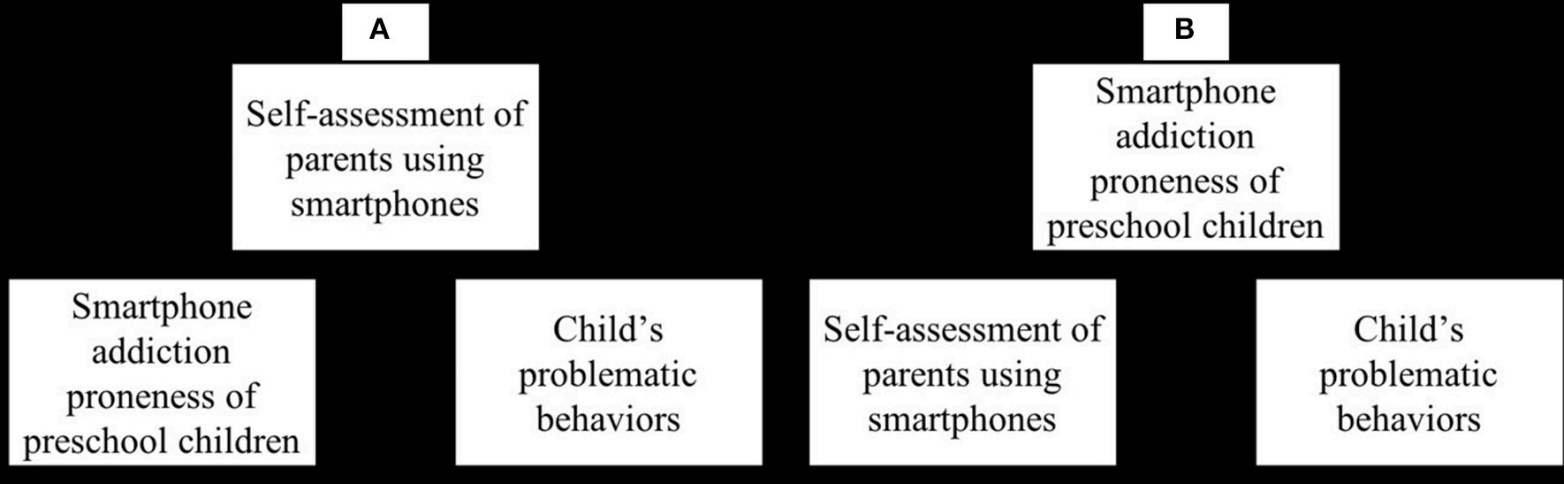
**(A)** depicts Cho and Lee's mediation model. **(B)** depicts an alternative mediation model.

Moreover, the psychometric properties of some of the scales administered in the study are problematic. First, the scale used to assess smartphone addiction proneness in pre-schoolers was originally developed as a diagnostic scale to screen adolescents (aged 10–17) and adults for risk of smartphone addiction (Shin et al., 2011). Thus, the scale may not accurately capture young children's smartphone addiction proneness. For example, items that measure the influence of smartphone addiction proneness on school performance or being scolded by teachers for frequent smartphone use might be valid behaviors to assess in adolescents, but not in infants or toddlers. Second, Cho and Lee's (2017) assessment of emotional intelligence (EI) in young children aged 1–7 is problematic, because EI has been used to predict positive social and academic outcomes only in children aged 3 and above (Izard et al., 2001; Denham et al., 2003)—that is, when children are able to independently regulate their emotions, develop specific coping strategies for different contexts, and rely less on external support from caregivers (Denham, 2007). Third, the smartphone-related questionnaires for both children and parents were reported by the same parent (either the mother or father), which raises the issue of common method variance—i.e., the degree to which correlations or covariance shared among variables are inflated due to use of the same measurement method. Given that common method variance could cause spurious relationships by biasing the actual relationship between the underlying constructs of interest (Lindell and Whitney, 2001), more rigorous sampling methods and techniques should be used to reduce potential common method problems. For the reasons addressed above, Cho and Lee's results should be interpreted with caution.

In conclusion, although Cho and Lee (2017) put forth an interesting idea, more work is warranted to verify the proposed model and psychometric properties of the measures. Further studies should employ concrete and objective measures of smartphone use to reduce issues associated with self-reports that are retrospective, potentially biased, and often inaccurate (Boase and Ling, 2013). More comprehensive longitudinal studies are necessary to advance our understanding of the impact of young children's smartphone use on various aspects of development in early childhood. In particular, the impacts of specific educational apps or the purposeful use of smartphones for learning deserve special attention, because they may have a beneficial influence, to different extents, on young children's cognitive development (Lieberman et al., 2009), such as executive functioning, which refers to a group of higher-level cognitive abilities that allow for flexible and complex goal-directed behaviors (Miyake et al., 2000). For example, a recent study suggests that children's working memory—a major aspect of executive functioning—improved after playing with an educational app compared to watching a cartoon (Huber et al., 2018). Moreover, given that media can influence one's emotion regulation (e.g., prolonging a positive mood; Greenwood and Long, 2009), future studies should also examine the impact of smartphone use on young children's emotional regulation and social competence, which would shed light on the socioemotional development of young children (Cole et al., 2004; Denham, 2007).

## Author Contributions

TQ, AH, WT, and HY contributed to discussion and writing the manuscript.

### Conflict of Interest Statement

The authors declare that the research was conducted in the absence of any commercial or financial relationships that could be construed as a potential conflict of interest.
